# The anterior LICAP flap: a design option for oncoplastic breast reconstruction 

**DOI:** 10.1080/23320885.2021.1986048

**Published:** 2021-10-01

**Authors:** Juliëtte E. D. Jacobs, Sanharib Al Shaer, Ute Schmidbauer, Daniëlle M. de Leeuw, Hinne A. Rakhorst, Oliver T. Zöphel

**Affiliations:** a Department of Plastic, Reconstructive and Handsurgery, Ziekenhuis Groep Twente (ZGT), Hengelo, The Netherlands; b Department of Surgery, Ziekenhuis Groep Twente (ZGT), Hengelo, The Netherlands

**Keywords:** Breast cancer, breast conserving surgery, oncoplastic breast reconstruction, anterior LICAP flap

## Abstract

The purpose was to describe the operation technique of an anterior lateral intercostal artery perforator (LICAP) flap and analyse outcomes and complications. An anterior LICAP flap is a good and safe alternative for direct oncoplastic breast reconstruction. It is a reliable flap that provides sufficient volume and good esthetic outcomes.

## Introduction

Breast cancer is the most common form of cancer in women worldwide. In the Netherlands, one in eight women (12.5%) develops breast cancer with 17.201 new cases in 2018 [1]. Early detection and treatment of breast cancer have improved over the past few years, leading to a better survival rate [2]. There have been dramatic changes in the surgical management of breast cancer in the twentieth century [3].

Breast-conserving surgery (BCS), followed by radiotherapy, has been shown to be equivalent to mastectomy in terms of oncological survival and superior patient-reported outcomes (PROs) [4,5]. BCS shows higher patient satisfaction and better cosmetic outcomes with fewer complications [5]. For these reasons, BCS had become the preferred surgical therapy in the treatment of early-stage breast cancer.

However, BCS had its limitations. When resection volumes rise, it may lead to a malformed breast shape [6]. Oncoplastic breast-conserving surgery (OPBCS) was developed to be able to resect larger tumors while maintaining optimal functional and cosmetic outcomes [7]. There are two main types of OPBCS; volume displacement, which includes using the remaining tissue of the breast to fill the defect, and volume replacement, which includes reconstruction of the breast with the transposition of tissue from elsewhere [7], such as the use of the lateral intercostal artery perforator (LICAP) flap.

Especially in volume replacement strategies, various donor sites have been used. The anterior LICAP flap is an addition to the current armamentaria for the OPBCS. This surgical technique uses a pedicled perforator flap to fill the defect of the lumpectomy. The hypothesis is that defects in all four quadrants can be reconstructed with an inconspicuous donor site. The purpose of this study was to describe the operation technique of an anterior LICAP flap and share the results of a retrospective analysis of outcomes and complications.

## Methods

### Patient selection and data collection

In this retrospective, descriptive study, all patients that underwent an anterior LICAP flap for oncoplastic breast reconstruction between February 2016 and June 2019 were included. In all patients, preoperative images were made. This study was approved by the regional Medical Ethics Committee.

Patients’ medical records were retrieved for the patient- and surgical characteristics and data was collected and stored in SPSS Statistics (version 24.0) in a pseudonymous manner. Patient characteristics included age, BMI, comorbidities, smoking status and previously received oncological therapy.

Operative details were collected. Data included the tumor location, tumor size and tumor weight. The localization of the tumor was classified by the four quadrants of the breast; lower-inner quadrant (LIQ), lower-outer quadrant (LOQ), upper-inner quadrant (UIQ), and upper-outer quadrant (UOQ). The tumor size, determined by preoperative MRI images, is presented in millimeters (mm) and the tumor weight, resected and weighed by the oncological surgeon, is presented in grams.

Patients were admitted on the day of surgery. Patients stayed in the hospital overnight. Drains were removed the next day. Patients were followed up using the regular local schedule. Postoperative images were made 3 months after surgery.

Postoperative complications were retrieved from medical records up to six months postoperatively. The Clavien–Dindo classification was used for categorizing complications [8]. A complication was defined as any complication at the recipient or donor site including wound dehiscence, infection, seroma, fat necrosis, re-exploration and flap loss.

### Statistical analysis

Descriptive statistics were used to report the baseline characteristics of the study. Mean values with standard deviation (SD) were used for continuous variables with a normal distribution. Frequencies and percentages were used for categorical variables. All statistical analyses were performed with Statistical software SPSS (Version 24.0, IBM Corp., Armonk, NY).

## Anatomy and preoperative landmarks

**Figure 1.**
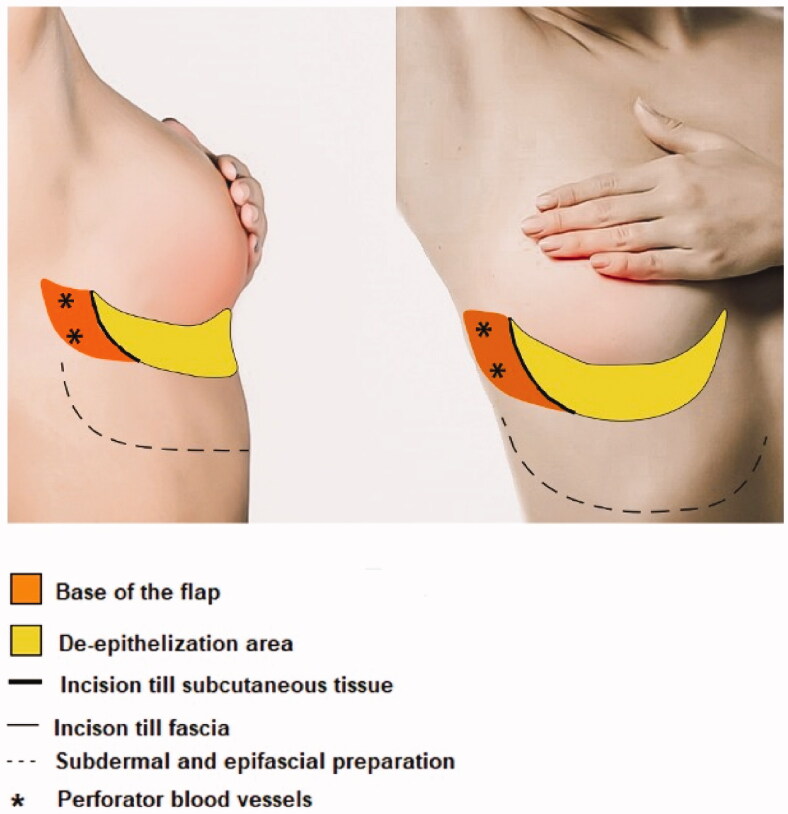
Design of the flap: lateral and frontal view.

The anterior LICAP flap is a flap based on the perforating arteries, which originate in the coastal segment of the intercostal arteries. It is an axial pattern flap based on the lateral intercostal artery perforators. Using a Doppler probe, one of two perforators of the lateral intercostal arteries are localized, and the flap is drawn onto the skin. The flap has a pivot point in the anterior axillary line. The cranial border is the inframammary fold (IMF). The width of the flap depends on the required volume and the laxity and surplus of the epigastric fat and skin. The width to length ratio is up to 1:4. Prior to surgery, the perforators, the design of the flap and the localization of the tumor were marked in standing position. The design of the flap is shown in Figure 1.

## Surgical technique

**Figure 2.**
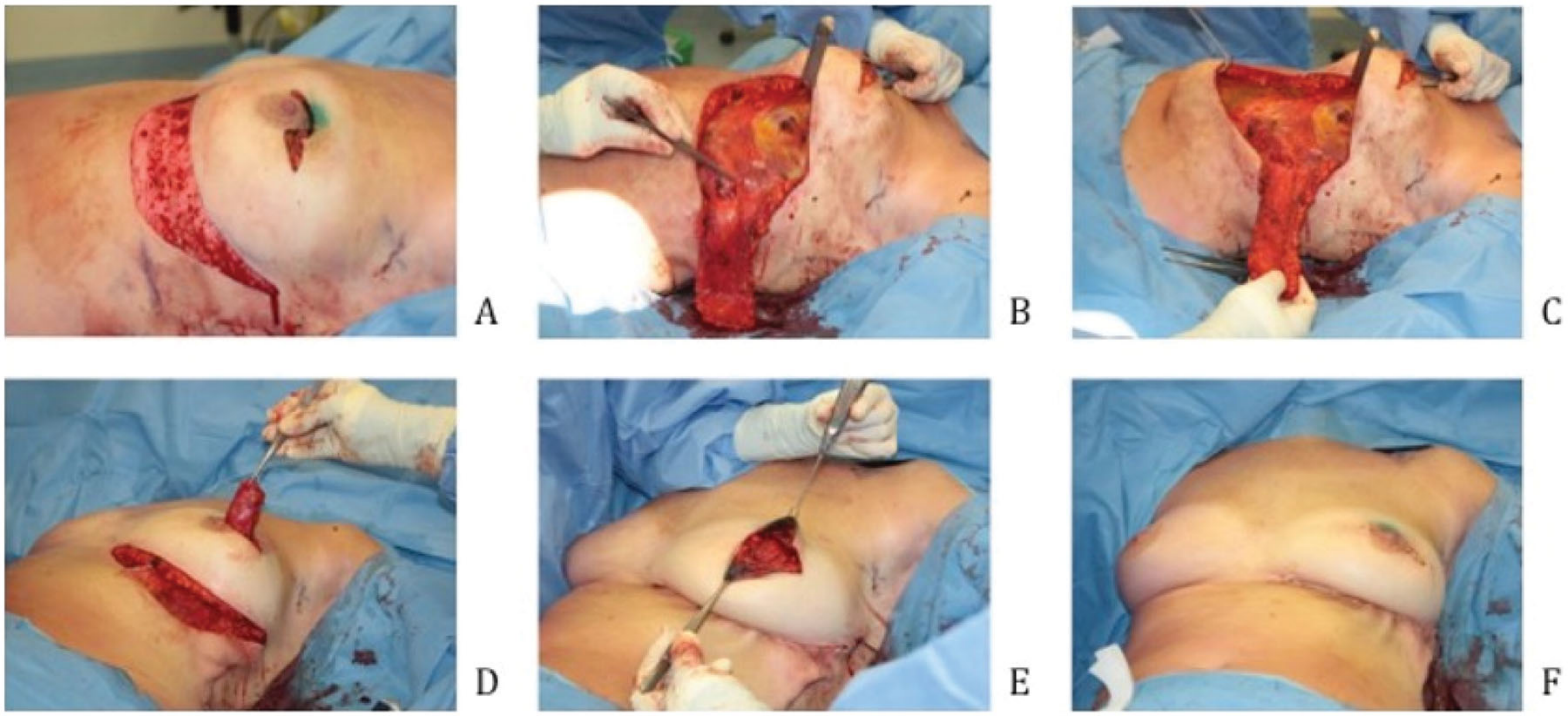
Surgical technique (A) Deepithelialization of the skin (B) Elevation of the subcutaneous adipose flap, saving the perforator (C) Subdermal preparation of the epigastric area (D) Tunneling of the flap (E) Fixation of the flap (F) Refixation of the IMF.

Figure 2 illustrates an overview of the surgical technique by intraoperative digital pictures. All patients received prophylactic intravenous antibiotics. The patients were positioned in a supine position with sterile dressings. The oncological surgeon performs a lumpectomy, and the specimen is weighed. Prior to flap dissection, the size of the defect is measured, and the flap is re-determined to fit the defect. The incision is made according to the preoperative design and intraoperative modifications. The flap is elevated over the fascia as a standard adipocutaneous perforator flap. The perforator typically does not need to be dissected. Then de-epithelialization of the flap is done apart from the tip of the flap to determine its blood supply after transposition. The flap is put into its position during donorsite closure. Donor site closure is done by epifascial undermining of the skin of the epigastric area towards the caudal, after which the IMF is reconstructed with a running PDS 1. Mobilizing the epigastric skin area over the fascia is necessary to close the donorsite and reconstruct the IMF without tension. The skin is closed in a standard fashion. Two suction drains are placed. After the closure of the donorsite the flap circulation is reevaluated by looking at the skin island at the tip of the flap. If perfusion is sufficient the skin island is removed, and the flap fixated. Fixation of the flap is done with Vicryl 1.0.

## Results

### Patient characteristics

From February 2016 until 19 August 19 modified lateral pedicled IMF flaps for oncoplastic breast reconstruction were performed. After the reconstruction, 89.5% received radiotherapy. The mean age was 59 years (SD 9.9), mean body mass index (BMI) was 24 (SD 3.3). All patient characteristics are presented in detail in Table 1.

**Table 1.** Patient characteristics.

Patients (*n*)	19
Mean age, years (SD)	59 (9.9)
Mean BMI (SD)	24 (3.3)
Diabetes mellitus, % (*n*)	5.3 (1)
Hypertension, % (*n*)	10.5 (2)
Smoking, % (*n*)	21.1 (4)
Anticoagulants, % (*n*)	10.5 (2)
Radiotherapy, % (*n*)	89.5 (17)
Chemotherapy, % (*n*)	36.8 (7)
Hormone therapy, % (*n*)	42.1 (8)

### Operative details

Tumor characteristics are presented in Table 2. The mean size of the tumor was 26 mm (SD 9.9) and the mean weight of the tumor was 80 grams (32.1). The localization of the tumor was classified by the four quadrants of the breast. Fourteen patients (74%) had a tumor in the upper-outer quadrant.

**Table 2.** Tumor characteristics.

Patients (*n*)	19
Mean tumor size, mm (SD)	26 (9.6)
Mean tumor weight, gram (SD)	80 (32.1)
Location tumor, % (*n*)	
Lower-inner quadrant	10.5 (2)
Lower-outer quadrant	10.5 (2)
Upper-inner quadrant	5.3 (1)
Upper-outer quadrant	73.7 (14)

### Complications

When using the Clavien-Dindo Classification for postoperative complications, this study shows that complications requiring surgical intervention (Grade 3), life-threatening complications (Grade 4) and mortality (Grade 5) did not occur (Table 3). One patient had wound dehiscence lateral to the areola that healed by secondary intention. One patient had an infected hematoma with the need of outpatient clinic drainage and one patient developed infected seroma with the need of antibiotics. All flaps survived. Delay to the start of adjuvant radiation therapy occurred in 5.3% of the cases.

**Table 3.** Complications categorized according to the Clavien–Dindo classification.

Patients (*n*)	19
Clavien–Dindo Classification, % (*n*)	
Grade 1	10.5 (2)
Grade 2	5.3 (1)
Grade 3	
- a	0 (0)
- b	0 (0)
Grade 4	
- a	0 (0)
- b	0 (0)
Grade 5	0 (0)

### Pre- and postoperative images

**Figure 3.**
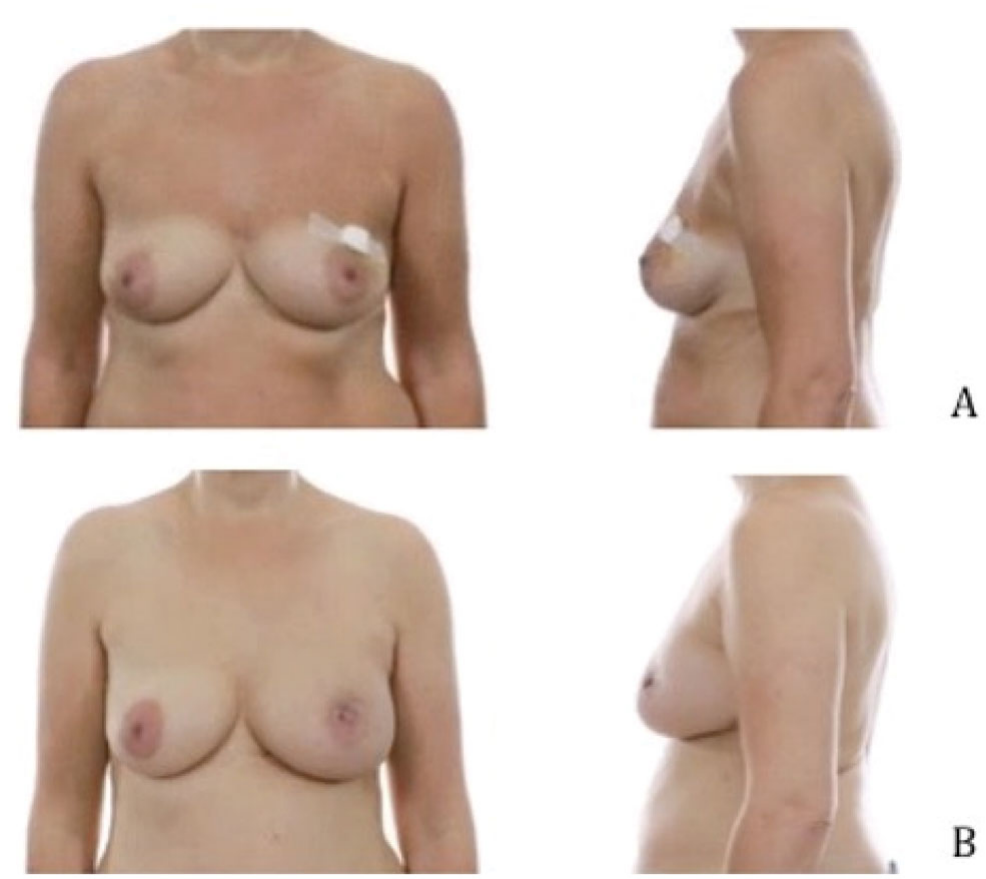
Location of the tumor: left upper-outer quadrant, size of the tumor: 27 mm, weight of the tumor: 75 gram (A) Preoperative frontal and lateral view (B) Postoperative frontal and lateral view.

**Figure 4.**
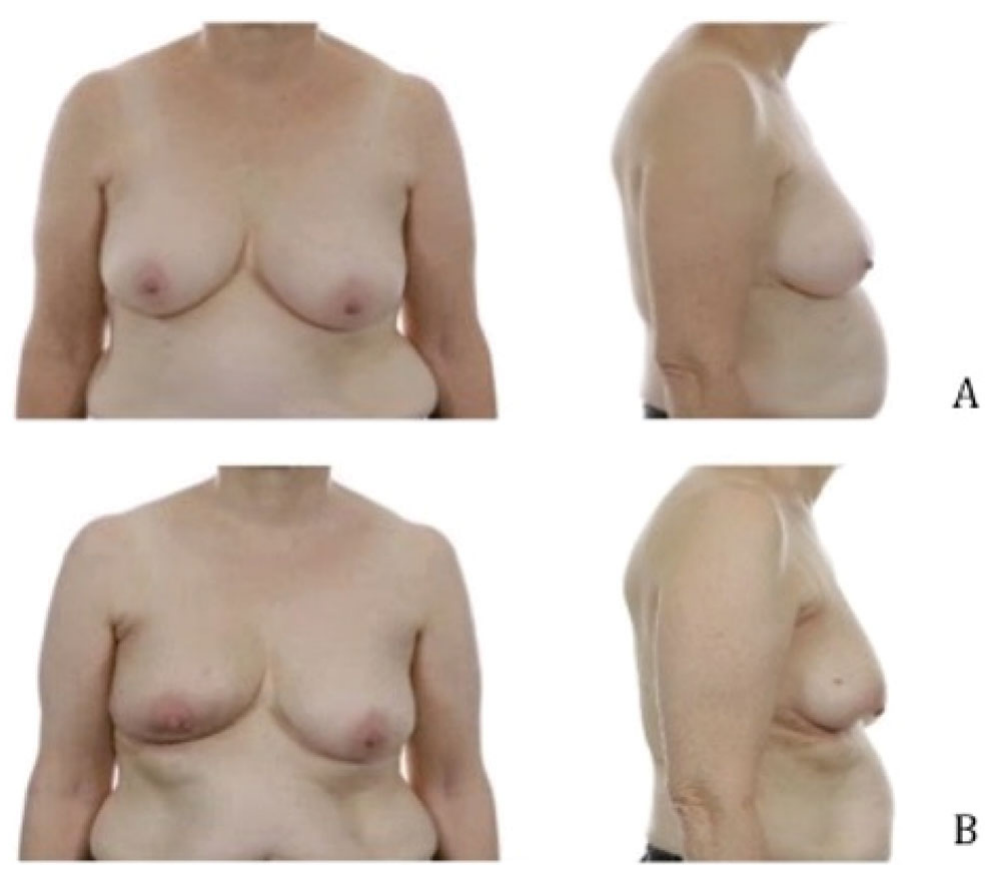
Location of the tumor: right lower-inner quadrant, size of the tumor: 16 mm, weight of the tumor: 51 gram (A) Preoperative frontal and lateral view (B) Postoperative frontal and lateral view.

**Figure 5.**
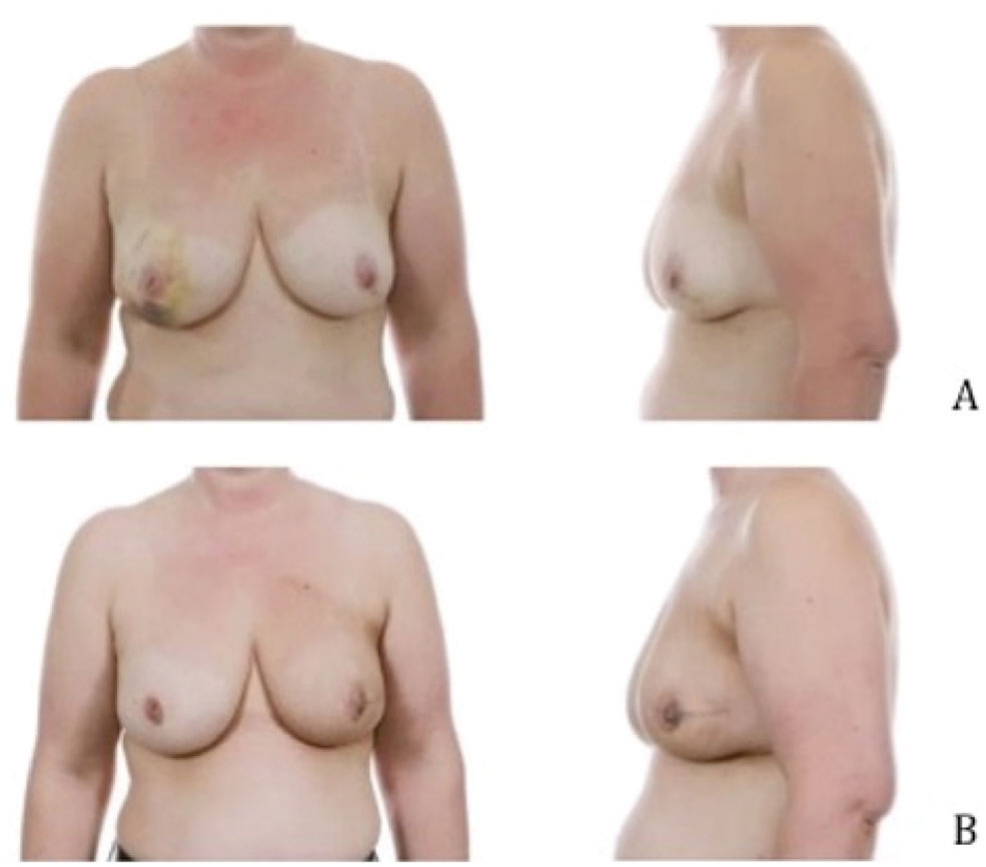
Location of the tumor: left upper-inner quadrant, size of the tumor: 28 mm, weight of the tumor: 90 gram (A) Preoperative frontal and lateral view (B) Postoperative frontal and lateral view.

Pre- and postoperative images were made to show the cosmetic results of the modified lateral IMF flap for oncoplastic breast reconstruction. Figures 3, 4 and 5 show the results of three patients. All patients had a different localization of the tumor. Image A is the preoperative status, B shows the postoperative result. The postoperative videos added to the article show online moving images of the three patients (Videos 1–3).

## Discussion

BCS has become the preferred surgical therapy in the treatment of early-stage breast cancer due to the dramatic changes in the surgical management of breast cancer in the twentieth century [3,4]. The optimal oncological outcome of BCS entails complete excision of the tumor with negative resection margins while maintaining satisfactory cosmetic results [9,10]. Tumor location and resection volume of the tumors has been proven to affect cosmetic outcome [11]. OPBCS was developed to deal with larger defects while maintaining optimized functional and cosmetic outcomes [7]. This study analyses the experiences of the anterior LICAP flap as a volume replacement technique for OPBCS. The purpose of this study was to describe the operation technique of an anterior LICAP flap and share the results of a retrospective analysis of outcomes and complications.

Many volume replacement techniques are described. Flaps are chosen based on defect size, location, surgeons’ experience and local habits. The anterior LICAP flap has potential use in breast reconstruction as an addition to the current armamentaria for OPBCS, such as the lateral intercostal artery perforator (LICAP) flap, the thoracodorsal artery perforator (TDAP) flap and the latissimus dorsi (LD) flap. The LICAP flap is based on perforators arising from the costal groove and is most suitable for lateral and inferior breast defects [12–14]. The TDAP and LD flap, which are based on the perforators from the descending or horizontal branches of the thoracodorsal vessels are most suitable for superior defects [9,14–16].

The anterior LICAP flap was developed to fill defects central or in the LOQ of the breast. Additionally, the IMF is an inconspicuous donor site with no functional impairment.

The results of the study show that defects in all quadrants of the breast can be filled, 73.7% of the tumors were located in the UOQ. The anterior LICAP flap is a reliable flap with only minor complications. Complications were treated according to the national guidelines with good results. Delay to the start of adjuvant radiation therapy occurred in one patient due to wound dehiscence that healed by secondary intention. Pre- and postoperative images show good esthetic outcomes and projection of the breast, with no functional impairment. One drawback of the flap is that flap is not suitable for all defects in the breast. The limiting factor is tumor size and patients’ surplus of epigastric fat and skin. The IMF is an inconspicuous donor site, but the retraction of tissue in the IMF has been taken into account.

Data on the use of the anterior lateral intercostal artery perforator flap for immediate breast reconstruction is limited. The study by Carrasco-Lopez et al. suggests that it is suitable for partial breast reconstruction and does not appear to negatively impact patient satisfaction [17]. However, the LICAP flap, the TDAP flap and the LD flap have been widely described. Meybodi et al. and Hakakian *et al.* both describe that the LICAP flap was mostly used for a tumor in the LOQ [13,18]. Advantages are no muscle sacrifice, flap reliability and low donor site morbidity. A limitation is that the scar that results is visible as it extends from the lateral mammary fold to approximately 3-4 cm posterior to the posterior axillary line [13]. Previous literature showed that the TDAP flap was frequently used for superior defects of the breast [16]. Abdelrahman et al. describe that with the LD and TDAP flap, 45.2% of the tumors were located in the UOQ [9]. The TDAP flap is as reliable as the LD flap regarding the achievability, postoperative complications and PROs with the better functional outcome of the shoulder [9]. Although, the LD flap can fill larger defects [9]. The studies by Koh et al. and Lee et al. concluded functional impairment in the form of postoperative arm and shoulder disabilities in clinical practice for both TDAP and LD flap [19,20]. Hamdi et al. describe that donor-site morbidity after harvesting a TDAP flap was reduced to a minimum [21].

Complications found in this study meet the results of peers. An average complication rate of 16% for minor and major complications is acceptable in OPBCS [10]. Most complications described in the literature are infection, seroma, delayed wound healing and flap loss. Long-term complications are breast fibrosis and asymmetry [10].

The results of this study should be interpreted while considering its limitations. First, the retrospective nature of this study makes it susceptible to recall or information bias. Second, the study describes a small group of patients with a minimum follow-up of 6 months postoperatively. Last, this study did not consider PROs. In future research, it would be valuable to include more patients with a longer duration of follow-up and to include PROs with a validated and breast cancer-specific questionnaire such as BREAST-Q.

## Conclusion

An anterior LICAP flap is a good and safe volume replacement technique for the immediate filling of defects following OPBCS. It is a reliable flap that provides sufficient volume with good esthetic outcomes and projection of the breast.

## Supplementary Material

Supplemental Material
