# Supplementary figures and images for: The anterior LICAP flap: a design option for oncoplastic breast reconstruction
Source: Case Reports Plast Surg Hand Surg. 2021 Oct 1;8(1):158–63. doi: 10.1080/23320885.2021.1986048 (PMC8491666; doi:10.1080/23320885.2021.1986048)

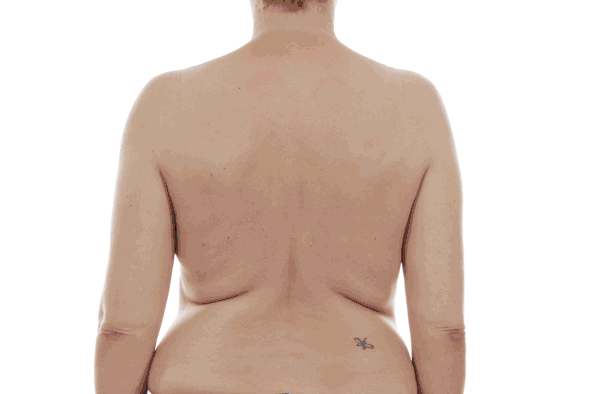

Supplement: Supplemental Material [file ICRP_A_1986048_SM3689.zip › suppl_videos/Video1.gif]

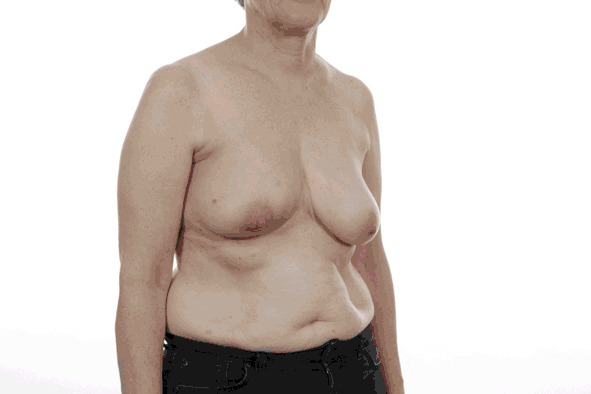

Supplement: Supplemental Material [file ICRP_A_1986048_SM3689.zip › suppl_videos/Video2.gif]

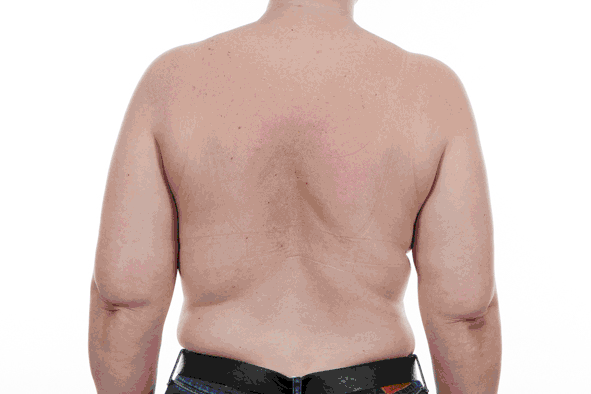

Supplement: Supplemental Material [file ICRP_A_1986048_SM3689.zip › suppl_videos/Video3.gif]
